# Netrin Family: Role for Protein Isoforms in Cancer

**DOI:** 10.1155/2019/3947123

**Published:** 2019-02-24

**Authors:** Caroline Suzanne Bruikman, Huayu Zhang, Anneli Maite Kemper, Janine Maria van Gils

**Affiliations:** ^1^Amsterdam UMC, University of Amsterdam, Department of Vascular Medicine, Location Meibergdreef, Amsterdam, Netherlands; ^2^Leiden University Medical Center, Department of Internal Medicine, Einthoven Laboratory for Vascular and Regenerative Medicine, Leiden, Netherlands

## Abstract

Netrins form a family of secreted and membrane-associated proteins. Netrins are involved in processes for axonal guidance, morphogenesis, and angiogenesis by regulating cell migration and survival. These processes are of special interest in tumor biology. From the netrin genes various isoforms are translated and regulated by alternative splicing. We review here the diversity of isoforms of the netrin family members and their known and potential roles in cancer.

## 1. Introduction

Alternative splicing is the process where regions of pre-mRNA molecules are joined or skipped in different ways, resulting in different protein coding transcripts [1]. This way biodiversity of proteins is much increased from a certain amount of protein coding genes. Alternative splicing can be part in the physiological process such as development of the brain [2], while it can also be associated with disease including Parkinson's disease and Schizophrenia [3, 4]. Changes in alternative splicing in tumors are detected more and more. These abnormal alternative splicing events may interfere with normal cellular homeostasis and be important signatures for tumor progression and therapy [5–10].

The netrin family is a class of highly conserved proteins which together with protein families such as semaphorins, slits, and ephrins make up the neuronal guidance cues [11]. They were originally discovered to play an important role in development of the central nervous system, but over the last decades they have been shown to involve in many more processes beyond their roles in the central nervous system, and, among them, a pivotal role in cancer [12–16]. So far, expression of six different netrin family members has been described in mammals.

**Figure 1:**
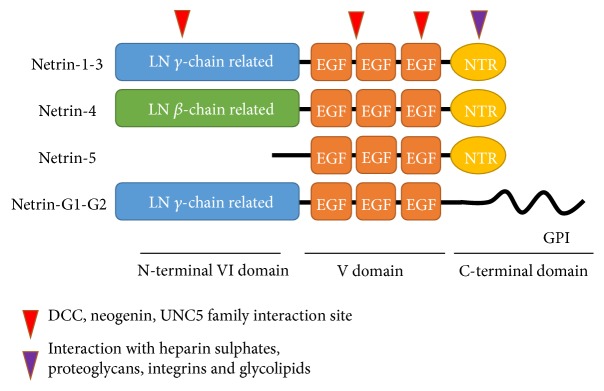
Schematic domain architecture of the netrins depicting a laminin-like (LN) domain, three epidermal growth factors like repeats (EGF), and the C-terminal domain (NTR). Arrow heads indicate interaction sites with the various netrin receptors or extracellular matrix proteins.

Netrin-1, netrin-3, netrin-4, and netrin-5 are secreted proteins; netrin-G1 and netrin-G2 are membrane bound proteins tethered by glycosyl phosphatidylinositol (GPI) anchors (see Figure 1) [17–19]. Netrin-2, an orthologue of netrin-3, only exists in worms and birds [20]. Although the different netrin proteins share a name, their homology is different. The N-terminal V and VI domains of netrin-1 and netrin-3 are similar to the laminin-*γ*1 chain, whereas the N-terminal end of netrin-4 shares homology with the laminin-*β*1 chain. Netrin-G1/2 has homology to both the *γ*1 and *β*1 chains, but is most homologous to the laminin-*γ*1 chain [21]. Due to the differences in homology between the netrin proteins, the receptors they bind to also varies between the different forms. Of all netrins, netrin-1 expression and function are most studied, since its orthologues are present and play a highly conserved role in all bilaterally symmetrical animals studied so far. Netrin-5 is the newest form of netrin identified in mammals [22]. In addition to their full length isoforms, various other isoforms have been described of various netrins. In this review an overview of the possible effect on ligand-receptor interactions and the role in cancer will be discussed for each netrin type and their described isoforms.

## 2. Various Roles for Netrin-1 in Cancer

Netrin-1 (NTN1) is the first described and most extensively researched from all netrins. It was first discovered in mammals in the 1990s [23]. Netrin-1 specifically guides axons to their final location leading to the formation of synapses through a balance of chemoattractive and chemorepulsive signals and does this by the guidance of the neuron growth cone, which is located at the tip of growing axons. In the last decade, NTN1 was found to be involved in many more processes in and beyond the nervous system, ranging from angiogenesis to inflammation [17, 24–27].

Netrin-1 ligand-receptor interactions have been shown to be important cell survival and tumorigenesis. Netrin-1 acts as an oncogene, and its upregulation was found in several cancers, including metastatic breast cancer, non-small-cell lung cancer, neuroblastomas, and pancreas adenocarcinomas [14, 28–31]. Tumor cell migration stimulated by NTN1 was seen in melanoma, glioblastoma, and pancreatic adenocarcinomas [30, 31]. It is suggested that angiogenesis can be stimulated by NTN1 in the presence of CD146 [32]; however CD146 as a receptor for NTN1 needs to be confirmed. Endothelial specific CD146 knockout mice do not show severe morphological vascular defects, but in a xenograft tumor model, reduced tumor volume and vascular density were observed [33]. These data suggest that this NTN1 affected adhesion molecule CD146 may play a role in pathological rather than physiological angiogenesis. The role of NTN1 in angiogenesis is still unclear at the moment.

Netrin-1 is also expressed by macrophages where it has immunomodulatory functions. Notably, NTN1 potently blocks directed migration of macrophages [34]. Tumor-associated macrophages (TAMs) also contribute to many steps of tumorigenesis, such as transformation, tumor cell proliferation, angiogenesis, invasion, and metastasis [35]. Whether NTN1 also blocks migration of TAMs and therefore contributes to the multiple steps of tumorigenesis is speculative, but an interesting pathway that needs additional studies.

A link between inflammation and cancer was found in NTN1 expression in some colorectal cancers. It was proposed that inflammation-induced NF*κ*B expression upregulates NTN1 expression which subsequently leads to reduced apoptosis and tumorigenesis [36, 37]. Netrin-1 receptors DCC (Deleted in Colorectal Carcinoma) and UNC5 act as ‘dependence receptors' and when surrounded by a NTN1 gradient, cell survival is prevailed. In absence of NTN1, gradient cell apoptosis is triggered both in vitro and in vivo [38–40]. The mechanism through which these receptors trigger apoptosis is currently unclear, but based on the crystal structure of the UNC5B intracellular domain, it is proposed that the proapoptotic domain of UNC5B is masked by NTN1. In the absence of the ligand, this domain is unmasked. The ability of these receptors to trigger apoptosis in the absence of their ligand has been proposed as a mechanism for tumor suppression [41]. An overexpression of NTN1 in mice leads to reduced apoptosis and to the formation of hyperplastic lesions and tumors [42, 43]. Tumor cells can turn the proapoptotic signalling off and maintain their survival by the loss of NTN1 receptors [44] or by increasing its NTN1 production and autocrine secretion which promotes tumor growth and metastases in several animal models [28, 45]. There is irrefutable proof in animal models of cancer that silencing NTN1 with small interfering RNA is associated with inducing cell apoptosis [28–30, 46]. Then the question remains as to the toxicity and side effects of drugs targeting NTN1, as it is known there are multiple roles for the NTN1 protein in different adult tissues. A significant hurdle to this is the lack of specific antibodies to target NTN1 specifically for pathological angiogenesis. Therefore, making a NTN1 a therapeutically target in pathological angiogenesis needs additional studies.

Interestingly and promising, no clinical, haematological, or biochemical signs of toxicity were noted in mice and monkeys who received a humanized anti-NTN1 antibody that disrupted the interaction between NTN1 and UNC5B. This triggered the death of NTN1 expressing tumor cells in vitro. Currently, the first clinical trial for NP137, a humanized monoclonal antibody targeting the NTN1 ligand, will assess the safety, tolerability, pharmacokinetic, pharmacodynamics, and preliminary antitumor activity in patients with locally advanced or metastatic solid tumors. This study will be completed in January 2020 and the results are eagerly awaited (www.clinicaltrials.gov, NCT02977195).

## 3. Netrin-1 Isoforms

**Figure 2:**
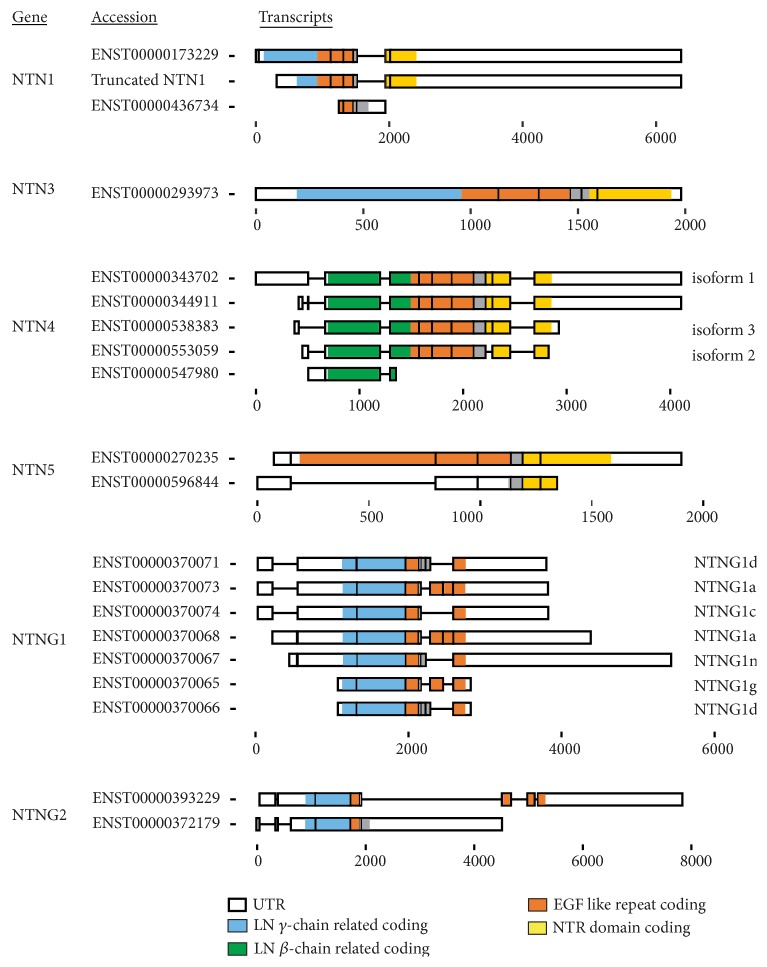
Schematic exonic composition of the alternative spliced netrin isoforms with evidence of protein translation. Accession numbers are Ensembl annotations. Colors indicate coding regions of the different protein domains. White boxes indicate untranslated regions. Numbers are of base pare scaling.

Although many aspects of NTN1 biology in cancer have been thoroughly investigated, the role of netrin isoforms is not well understood and could be helpful in designing therapy targeting pathological specific angiogenesis or cell survival. Netrin-1 is a secreted protein encoded by the NTN1 gene which is located on chromosome 17p13.1 in human genome. The full transcript consists of 7 exons and is translated into a 604 amino acid long protein. In some cancers a truncated form of NTN1 is detected and associates with poor patient survival [14, 47]. This truncated isoform is produced by an alternative internal promotor. As a result of the alternative start site, the truncated NTN1 lacks the first part of the N-terminal VI domain (Figure 2) and is shown to localize to the nucleolus. It interacts with nucleolar proteins and ribosomal DNA promotor sequences; this activity stimulates cell proliferation and acts protooncogenic in vitro and in vivo [14]. Passacquale et al. have shown that endothelial cells have increased expression of the nuclear localizing truncated NTN1 and reduced secretion of full length NTN1 upon treatment by the inflammatory stimulus TNF-*α* [48]. The transcription of the nuclear isoform is NF*κ*B dependent, and inhibition of NF*κ*B, as well as aspirin treatment, could prevent the transcription and localization to the nucleus of the truncated NTN1. Also it is not known whether truncated netrin isoforms can also bind the same receptor families as the common spliced netrins.

Next to this truncated NTN1 isoform, a potential isoform of NTN1 has been computationally mapped in the UniProt protein database [49]. This isoform is protein coding, resulting in a 153 amino acids protein containing part of domain V domain, namely, part of EGF-like repeats 2 and 3 (Figure 2). It thereby lacks both the N-terminal and C-terminal end of the full length protein, which could influence its ligand-receptor binding and in addition would make it more soluble due to the loss of interactions with extracellular matrix proteins.

## 4. Netrin-3

In humans, netrin-3 (NTN3) was first described in 1997 as netrin-2-like (NTN2L) protein because of its homology to the earlier discovered netrin-2 in chick embryos [50]. Netrin-3 is a 580 amino acid protein encoded by the NTN3 gene located on the human chromosome 16p13.3. In the literature and protein databases only one NTN3 isoform is described so far [50]. Like the other netrins, NTN3 is involved in the development of the nervous system [20, 51]. Netrin-3 has in structure a high similarity to NTN1 but has a lower binding affinity to the DCC receptor [20]. Little is known of NTN3 functions outside of the nervous system. One study reports that, in a search for biomarkers of acute kidney disease, NTN3 mRNA expression was moderately upregulated after reperfusion in ischemic kidney injury [52]. Also, mutations in the NTN3 gene have been reported in different databases to be associated with the development of several carcinomas [49]. The relative mRNA expression of NTN3 has been quantified in tumor and normal prostate tissue. A weak expression of NTN3 was observed, but no differences in expression between normal and neoplastic prostate tissues [53]. Nevertheless, additional studies are needed to understand the NTN3 function and possible isoforms present with respect to tumor biology.

## 5. The Role of Netrin-4 in Cancer

Netrin-4 (NTN4), also known as *β*-netrin, is one of the secreted netrins and was primarily discovered by its role in embryonic nervous system development where it controls proliferation and migration of adult neural stem cells destined for the olfactory bulb [54–57]. Netrin-4 shares a structural relation to the short arms of the laminin *β* chains and hence its other name *β*-netrin. This makes NTN4 different from the other netrins, since these show a structural homology to the short arms of laminin *γ* chains [55].

Besides its functions in the developing nervous system [55, 56, 58, 59], NTN4 has been identified to play a role in several other processes. Such that, for example, an inhibiting role for NTN4 was found in osteoclast differentiation, preventing bone loss in a mouse model [60]. Narrowing the scope to cancer, NTN4 has been found to be involved in the development and metastasizing of several kinds of cancer. It differs per cancer type whether NTN4 has a positive or negative association with disease progression. High NTN4 expression was reported in glioblastomas [61, 62] and in invasive breast tumors [63, 64]. The NTN4 expression in breast cancer tumors was correlated with longer disease-free survival and overall survival [64]. Netrin-4 expression was screened to be a biomarker in estrogen receptor *α* positive breast carcinomas, which are associated with favorable prognosis as well [65, 66].

In gastric cancer patients' tumor tissues and serum samples increased levels of NTN4 were, on the other hand, a biomarker correlated with a relatively poor survival rate. In gastric cancer NTN4 promotes the proliferation and motility of gastric cancer cells via the receptor neogenin1, which activates multioncogenic pathways [67]. Also neuroblastoma patients with high expression levels of both neogenin1 and NTN4 have a poor survival rate. The interaction between NTN4 and neogenin1 maintains the growth of neuroblastoma cells via prosurvival and promigratory molecular signalling [68]. In melanomas elevated levels of NTN4 played a functional role in the progression of metastasis and were proposed to be a potential target for therapy [69]. Netrin-4 also presents itself as a prolymphangiogenic factor; NTN4 overexpressing tumors in mice showed more metastases due to increased lymphatic permeability [70].

Considering proliferation and angiogenesis are important processes in carcinogenesis, it is of interest that NTN4 has a role in the regulation of endothelial proliferation. It has an inhibitory effect on the angiogenesis in endothelial cells in vitro [71]. It also inhibits the angiogenesis in the human placenta and could play a role in angiogenesis related pathologies during pregnancies [72]. Another study shows that knockdown of NTN4 increases vascular branching [73]. These and other studies combined indicate an inhibitory role for NTN4 on angiogenesis [74–76]. As for therapeutically targets, like the effects of NTN1, the role for NTN4 in cancer shows conflicting results. Whether the differences in experimental conditions could explain these contradictory results, or whether NTN4 should be blocked or stimulated is based on in what tissue the tumor occurs, has to be determined with further analysis.

## 6. Netrin-4 Isoforms

The gene that encodes NTN4 is in humans located on chromosome 12q22 and consists of 10 exons and 9 introns. In total five human NTN4 isoforms, produced by alternative splicing, have been described so far (Figure 2). The full length isoform of NTN4 is translated in a protein of 628 amino acids, which is the most described and most prevalent NTN4 form in both human and mice [55]. An alternative splice variant of NTN4 (isoform 3) has been identified by Zhang et al. [58]. They report that a large part of exon 1 is spliced out, resulting in the loss of the signal-peptide in the protein product of isoform 3 [58], which would prevent the secretion of the NTN4 isoform 3 protein. Another alternative spliced NTN4 mRNA is annotated in Ensembl containing similar coding to the protein isoform 3, but with different 5′UTR and 3′UTR. This NTN4 isoform 3 protein shows some similarities to the truncated NTN1 that is localizing to the nucleus [14]. Whether NTN4 isoform 3 protein localizes in the cytoplasm or nucleus and what its function is remain unknown. In addition, another isoform of NTN4 (isoform 2) is described in GenBank, no AY330211. This isoform lacks exon 8, which would result in a protein lacking part of the NTR domain. This might affect the ECM interaction or remodelling by NTN4. Last, an 176 amino acid long protein isoform, only containing the N-terminal VI domain, has been annotated in the UniProt protein database [49]. Given the pronounced role for NTN4 in tumor biology, the expression levels and regulation of NTN4 isoforms are of particular interest.

## 7. Netrin-5 Function and Isoforms

Netrin-5 (NTN5) is the most recently discovered netrin and has not been extensively studied thus far. Two studies investigating the role of NTN5 in mice have been published. One describes its expression and role in the adult brain [22], whereas the other study aimed for more understanding of the role of NTN5 in the developing nervous system [77]. In the developing nervous system NTN5 was found to be expressed on boundary cap cells, a transient neural-crest derived cell population located where the sensory axons enter and the motor neurons leave the spinal cord in the embryonic nervous system. There it prevents a disturbed migration of motor neuron cell bodies out of the ventral horn of the spinal cord [77]. In the adult mouse brain NTN5 was strongly expressed in neuroproliferative regions in the dentate gyrus of the hippocampus suggesting its involvement in adult neurogenesis [22]. As a small error during neurogenesis can initiate a cascade of reactions that may result in the formation of a glioblastoma [78], NTN5 can be of importance in malignant brain tumors specifically.

The gene for NTN5 is located on chromosome 19, location q13.33. Searching the Uniprot database [49] we found in total 2 human NTN5 isoforms described and 1 potential human NTN5 isoform that is computationally mapped (UniProtKB-Q8WTR8). Isoform 1 of NTN5 protein contains three EGF-like repeats and the NTR domain, but has a smaller N-terminal domain compared to the other netrins. Isoform 2 of NTN5 lacks the NTR domain [79]. Both these NTN5 isoforms might bind to the various netrin receptors and regulate cell migration and survival; however this has not been tested so far. The computationally mapped isoform only contains part of the C-terminal NTR domain and would be only 75 amino acid long upon translation. Since the quite recent discovery of this netrin, roles for NTN5 with its different isoforms in cancer are still to be eluded. Considering the role of NTN5 in cell migration it is well possible that NTN5 has a role in tumor biology.

## 8. Netrin-G1 Functions and Isoforms

Netrin-G1 (NTNG1) is one of the two GPI-linked, vertebrate specific netrins and is predominantly expressed on the presynaptic axonal side in neurons [21, 80]. During the embryonic development NTNG1 is responsible for the promotion of the neurite outgrowth of thalamic neurons via signalling of its receptor NGL-1 [81]. Also, NTNG1 is involved in the formation and maintenance of synaptic connections [82] and it contributes to the segment-specific differentiation of dendrites [83].

Netrin-G1 is only found in vertebrates and was first discovered in mice, where its gene, NTNG1, encodes for six isoforms formed by alternative splicing [21], regulated by the DNA/RNA-binding protein FUS (mutations in fused in sarcoma) [84]. In humans, the NTNG1 gene is located on chromosome 1 and contains 10 exons. Complex alternative splicing results in at least nine different mRNA isoforms of netrin-G1, plus a truncated alternative splice isoform [84, 85]; several have also been shown to be translated into protein (Figure 2). Netrin-G1a resembles in terms of structure the other netrins the most. Nerin-G1m is identical to NTNG1a except for additional inclusion of exons 6 and 7, making it the longest isoform. The additional two insertions, between the EGF-like repeats 1 and 2, are coding for sequence of 42 and 22 amino acids, respectively. Both of these insertions are not homologous to any known protein. All described NTNG1 isoforms, well described by Meerabux et al. [85], have various inclusion and exclusions of exons 5-9, ranging from the end of the EGF-like repeat 1 to EGF-like repeat 3. The only exception is the truncated isoform, which only contains domain VI and part of the first EGF-like domain [85], thereby lacking the GPI anchor resulting in a secreted protein upon translation. The spliced isoforms of NTNG1 are conserved among many species, which indicates a functional importance. The isoforms do have diversification in expression location and levels. Some isoforms only occur in adult tissue, whereas others are detected in both foetal and adult tissue. Human NTNG1d is mostly expressed in the kidney, but also in the foetal and adult brain, NTNG1a and NTNG1c mostly in the adult brain and NTNG1e in the foetal brain [85]. Although some functions of NTNG1 have been investigated, the potential role and regulation of NTNG1 isoforms in cancer have still to be uncovered.

## 9. Netrin-G2 Functions and Isoforms

Netrin-G2 (NTNG2) is the other GPI-linked vertebrate specific netrin. It was first described in mice [86]. Like NTNG1, NTNG2 is predominantly expressed in the brain, but some expression is also reported in the lung, kidney, heart, and spleen [80]. In the mouse brain NTNG2 expression is mainly located on axons in the cerebral cortex, habenular nuclei, and superior colliculus [86]. The NTNG2 domain structure resembles that of NTN1 and NTNG1a, an N-terminal VI domain, V domain with the 3 EGF-like repeats, and the C-terminal NTR domain. Similar to NTNG1, NTNG2 has a C-terminal domain containing a hydrophobic region with a GPI anchor [80, 86]. Roles for NTNG2 have been attributed to neuronal development. In an in vitro study with mutations induced in a gene (KDM5C) that encodes a histone demethylase involved in brain development and behavior, NTNG2 was downregulated compared to wild-type controls. This leads to a phenotype with suppressed neurite outgrowth and is restored upon NTNG2 overexpression in the mutant cells [87]. In studies where mice lack either NTNG2 or its receptor NGL2, normal auditory responsiveness is impaired, emphasizing both acting on the same neural development pathways in vivo [88].

In addition to the studies that are published about NTNG2 involving the function in brain development and behavior, a couple of studies have investigated NTNG2 outside of the nervous system and related to cancer. Netrin-G2 gene expression is found to be significantly reduced in superficial bladder transitional cell carcinoma [89] and upregulated in solid pseudopapillary neoplasms of the pancreas [90]. No research has reported on the function of the netrin-G2 protein in these cancers, and experiment to determine the functional consequences has to be executed before any statements about possible therapeutically potentials can be made.

In regard to different isoforms, NTNG2 has been studied very limited. Next to the full length isoform, alternative splicing for NTNG2 is suggested, so far 1 detected [85, 91] (Figure 2). This alternative spliced isoform lacks the EGF-like repeat 3 and the C-terminal domain with the GPI anchor, somewhat similar as the truncated NTNG1. Compared to NTNG1, NTNG2 has fewer exons, which could explain the fewer isoforms detected, but this needs still further exploration.

## 10. Conclusion and Future Perspectives

The amount of studies concerning netrins and their functions keeps on growing. The netrins are not only involved in axonal guidance, for which they have initially been identified. More and more evidence is emerging telling us that we can speak of the netrin family as general guidance cues instead of just neural guidance cues. We have seen involvement in tissue morphogenesis of different organs, immune cell guidance on top of axonal guidance, and angiogenesis in both noncancerous and cancerous tissues. The alternative splicing of netrin isoforms might play a main role in the diversity in guidance functions of the netrin family. Missing a domain or an extra domain could influence the binding affinity to receptors and connective tissue which subsequently has functional consequences. The most obvious would be different localization, intracellular, and extracellular, as we have seen nuclear localization of netrin-1 isoform. Moreover, multiple netrins isoforms are detected lacking the C-terminal domain, which would make the netrin more soluble due to less interaction with extracellular matrix proteins. Or in case of the NTNG1 and G2 soluble instead of membrane bound, an alternatively spliced netrin isoform could thereby get antagonistic functions compared to their full length variant. We have focused on the netrins, but have not taken into account possible splice isoforms of the different receptors. Additionally, this review has not taken into account alternative splicing of 3′UTR or alternative polyadenylation sites, which can affect gene expression levels. Furthermore, in addition to alternative splicing producing protein isoforms, variants in proteins can also be created by protein cleavage, as is, for example, described for NTN1, which can be cleaved by matrix metallopeptidase 9, generating domain VI-V fragments that have lower binding affinity to the UNC5B receptor and contribute to vascular permeability [92]. With the on-going discovery of new netrin family members and isoforms within those family members multiple questions about their function in cancer remain unclear. This review shows that little is known and much needs to be discovered regarding the (alternatively spliced) netrin family in cancer.
